# Solving the mystery of the FMC63-CD19 affinity

**DOI:** 10.1038/s41598-023-48528-0

**Published:** 2023-12-27

**Authors:** Jacqueline Seigner, Charlotte U. Zajc, Sarah Dötsch, Caroline Eigner, Elisabeth Laurent, Dirk H. Busch, Manfred Lehner, Michael W. Traxlmayr

**Affiliations:** 1 Department of Chemistry, Institute of Biochemistry, University of Natural Resources and Life Sciences, Vienna, Austria; 2 Department of Biotechnology, Institute of Animal Cell Technology and Systems Biology, University of Natural Resources and Life Sciences, Vienna, Austria; 3 CD Laboratory for Next Generation CAR T Cells, Vienna, Austria; 4 Institute for Medical Microbiology, Immunology and Hygiene, Technical University of Munich, Munich, Germany; 5 BOKU Core Facility Biomolecular and Cellular Analysis, University of Natural Resources and Life Sciences, Vienna, Austria; 6 St. Anna Children’s Cancer Research Institute, CCRI, Vienna, Austria; 7 Department of Pediatrics, St. Anna Kinderspital, Medical University of Vienna, Vienna, Austria

**Keywords:** Biochemical assays, Protein design, Surface plasmon resonance, Immunotherapy

## Abstract

The majority of approved CAR T cell products are based on the FMC63-scFv directed against CD19. Surprisingly, although antigen binding affinity is a major determinant for CAR function, the affinity of the benchmark FMC63-scFv has not been unambiguously determined. That is, a wide range of affinities have been reported in literature, differing by more than 100-fold. Using a range of techniques, we demonstrate that suboptimal experimental designs can cause artefacts that lead to over- or underestimation of the affinity. To minimize these artefacts, we performed SPR with strictly monomeric and correctly folded soluble CD19, yielding an FMC63-scFv affinity of 2–6 nM. Together, apart from analyzing the FMC63-scFv affinity under optimized conditions, we also provide potential explanations for the wide range of published affinities. We expect that this study will be highly valuable for interpretations of CAR affinity-function relationships, as well as for the design of future CAR T cell generations.

## Introduction

T cells genetically modified to express chimeric antigen receptors (CAR T cells) are among the most promising approaches in the field of cancer immunotherapy. In particular, CD19-reactive CAR T cells have yielded impressive response rates against hematologic malignancies such as B cell acute lymphoblastic leukemia (B-ALL) and diffuse large B cell lymphoma (DLBCL)^1^. Briefly, a typical second generation CAR molecule is composed of an extracellular antigen binding domain (usually a single-chain variable fragment, scFv)^2,3^, followed by a spacer region, a transmembrane domain and intracellular signaling domains derived from a costimulatory receptor (usually CD28 or 4-1BB) and from CD3ζ^1,4^. One of the most important parameters determining CAR function is its affinity to the target antigen. For example, it has been shown in several studies that the affinity determines the sensitivity of the CAR T cells, i.e., the required antigen density on the target cell. That is, T cells expressing low affinity CARs in the high nM or µM range require higher antigen densities to be properly activated^5–8^. In another study, CD19-reactive CAR T cells with an intermediate affinity scFv (*K*_D_ of 14 nM) showed higher proliferation and anti-tumor activities in vivo when compared with a high affinity CAR (*K*_D_ of 0.3 nM)^9^. Together, these studies demonstrate that CAR affinity is a crucial parameter determining CAR potency and antigen sensitivity.

**Figure 1:**
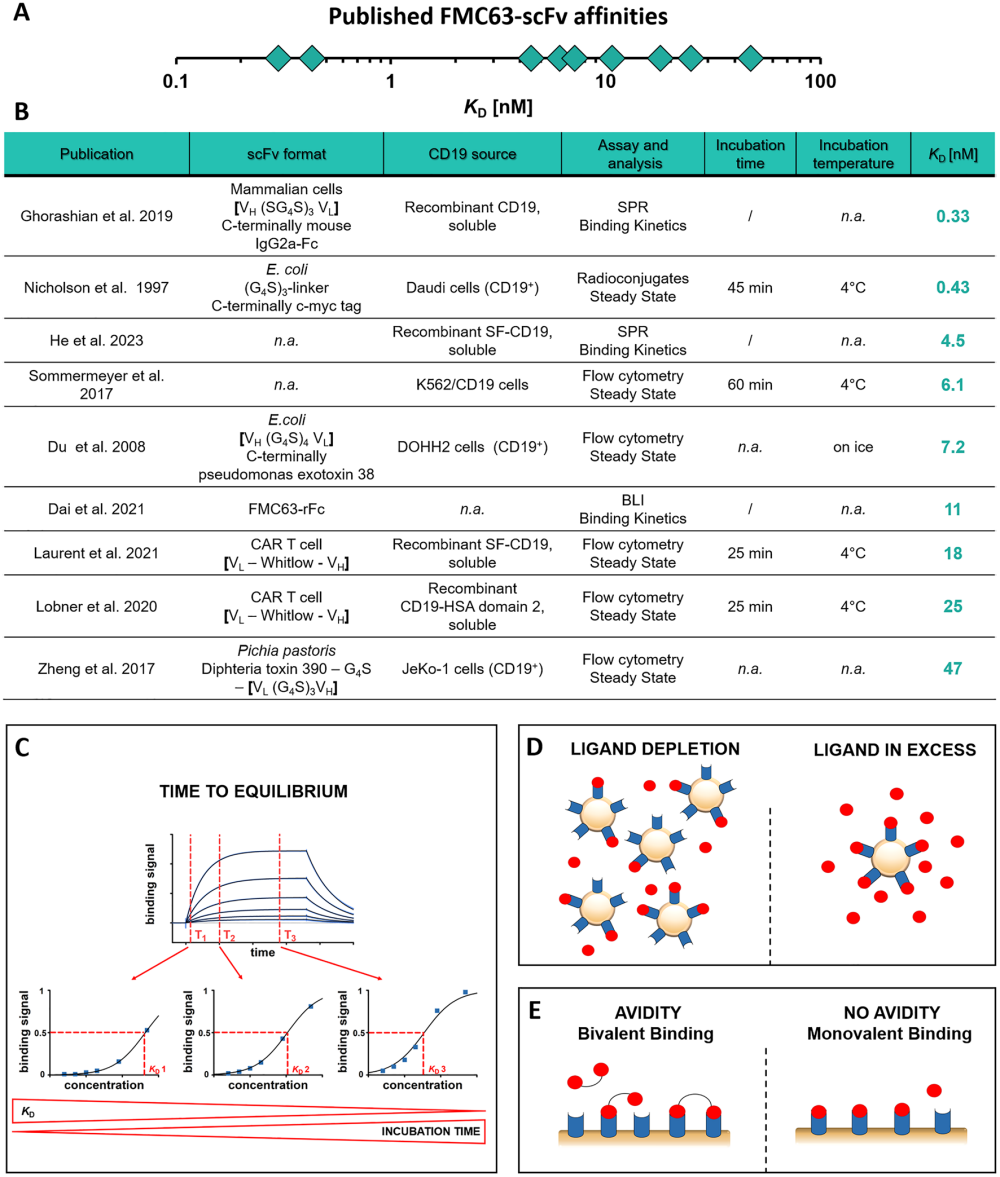
Summary of reported *K*_D_ values for the interaction of the FMC63-scFv with CD19 and potential artefacts in steady-state binding assays. (**A**) Overview of published FMC63-scFv affinities. (**B**) Table summarizing publications describing the interaction of the FMC63-scFv with CD19, including the source and architecture of the two interaction partners as well as the applied method to determine the *K*_D_ value; n.a., not available. (**C**–**E**) Illustrations explaining common artefacts and pitfalls when determining affinities by steady-state analysis.

Currently, CD19 is the most frequently targeted antigen in the CAR field^10^. Four out of six FDA-approved CAR T cell therapies are directed against CD19 and all of those are based on an scFv derived from the murine CD19-specific monoclonal antibody FMC63^11,12^. Moreover, FMC63-based CARs have been used as benchmarks in numerous preclinical studies^8,9,13–17^. Surprisingly, despite the high importance of the FMC63-scFv in the CAR field, inconsistent results regarding its affinity to CD19 have been reported in literature. In an extensive literature search, we found FMC63-scFv affinities ranging from 0.3 to 47 nM, thus differing by more than 100-fold (Fig. 1A,B)^5,9,11,16,18–22^.

Therefore, given the functional relevance of CAR affinity discussed above, the goal of the present study was a detailed affinity analysis of the FMC63-CD19 interaction. We show that suboptimal experimental designs cause artefacts that influence the obtained affinities, potentially explaining the wide range of affinities reported in literature. In particular, we demonstrate the impact of ligand depletion, insufficient equilibration times and avidity effects on the measured affinity. Finally, we designed experiments in which these artefacts have been excluded or minimized, yielding an FMC63-scFv affinity of 2–6 nM.

## Results and discussion

### Insufficient equilibration time and ligand depletion artificially decrease the measured affinity

Given the broad distribution of published FMC63-scFv affinities (Fig. 1A,B), we hypothesized that insufficient equilibration times and/or ligand depletion may be responsible for these inconsistencies. Both of these artefacts are known to influence the measured affinity in suboptimal experimental designs^23,24^. When measuring an affinity by steady-state analysis (usually done by titrating one of the two interaction partners), the system needs to sufficiently approach equilibrium. As a consequence, if the incubation is stopped too early, affinity is underestimated (Fig. 1C). Furthermore, when analyzing the affinity by ligand titration, it is assumed that the concentration of the ligand added to the reaction is similar to that after the equilibration phase. That is, consumption of ligand due to complexation should hardly affect the concentration of free ligand, which is only ensured if the ligand is present in large excess compared to its interaction partner expressed on the cell surface. In the absence of such an excess, a significant fraction of the ligand is depleted due to complexation, leading to artificially lowered ligand concentrations and underestimation of affinities (Fig. 1D). Importantly, both of these artefacts are mainly observed at low ligand concentrations in the low nM or pM range, which are typically required for analyzing high affinity interactions^23,24^.

Therefore, we tested whether incubation times and ligand depletion influence the measured FMC63-scFv affinity. In a first experimental system, we titrated the FMC63-scFv on the human CD19^+^ leukemia model cell line NALM6 using various incubation times, as well as different conditions that either promote or avoid ligand depletion. Indeed, we observed decreasing *K*_D_ values with prolonged incubation times indicating that the interaction has not reached equilibrium after standard incubation times of 0.5 or 1 h at 4 °C (Suppl. Fig. 1A).

**Figure 2:**
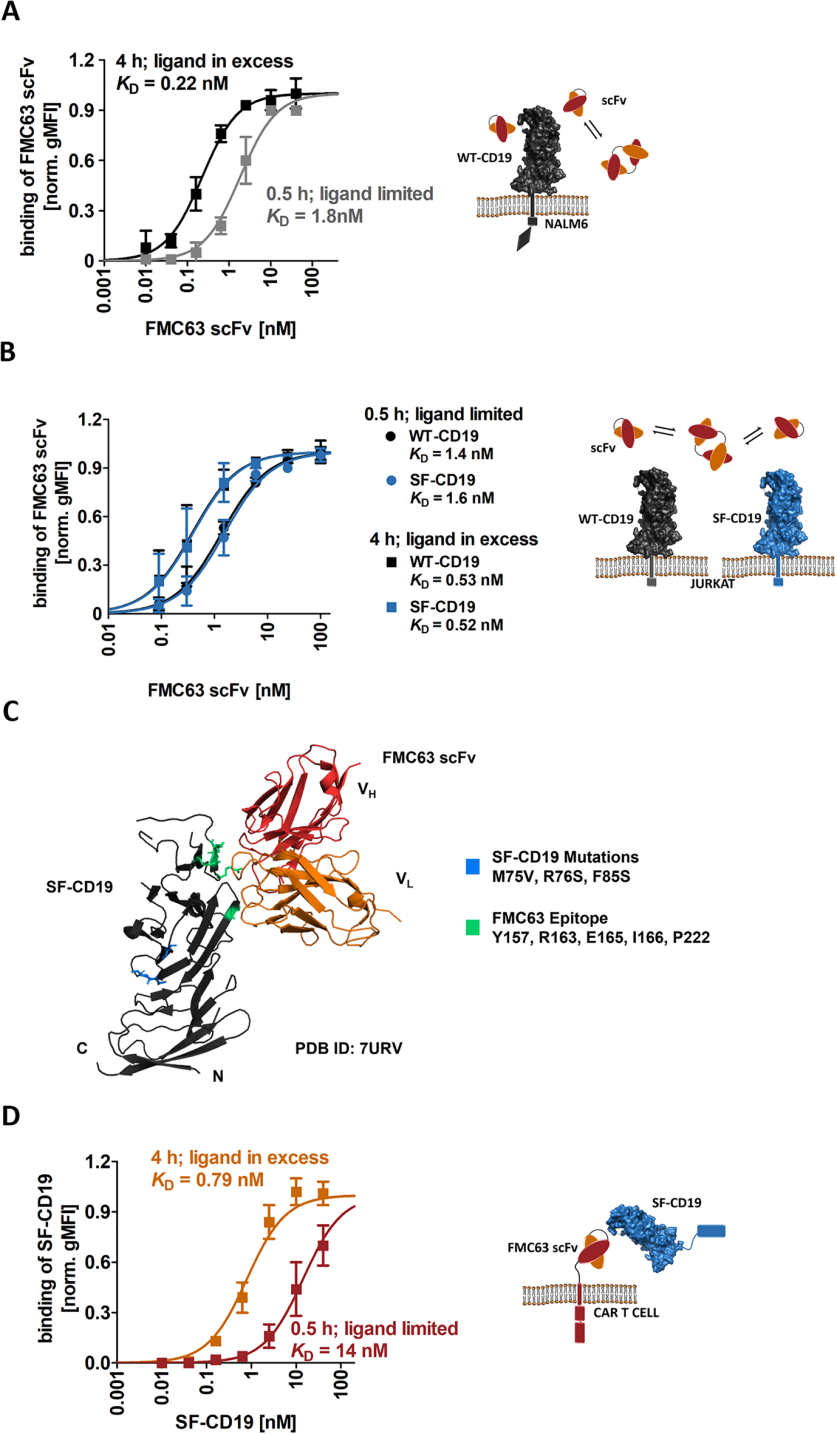
Determination of FMC63-scFv binding affinity to CD19 by steady-state analysis. (**A**) NALM6-GFP cells endogenously expressing WT-CD19 were incubated with various concentrations of His-tagged FMC63-scFv at 4 °C for either 0.5 or 4 h, as indicated, followed by staining with α-HIS-AF647 for subsequent flow cytometric analysis. (**B**) Titration of soluble FMC63-scFv on CD19 negative Jurkat cells transiently expressing Flag-tagged WT-CD19 or Flag-tagged SF-CD19. Incubations were performed with His-tagged FMC63 scFv at 4 °C for either 0.5 or 4 h. Secondary staining was performed with α-FLAG-PE and α-HIS-AF647. gMFI of FMC63-scFv binding of Flag-positive Jurkat cells was analyzed. Moreover, conditions were chosen to either promote or avoid ligand depletion, as indicated. (**C**) Representation of SF-CD19 in complex with the FMC63-scFv (PDB-ID 7URV) indicating in blue the three point mutations of the stability engineered SF-CD19 and in green the binding epitope of the FMC63-scFv on CD19. (**D**) Titration of SF-CD19 fusion protein (N-terminal His_8_-SUMO-tag) on FMC63:41BBz CAR expressing Jurkat cells. Secondary staining was performed with α-HIS-AF647. (**A, B** and** D**) Shown are averages ± standard deviations of background subtracted and normalized geometric mean fluorescence intensities (gMFIs) of three independent experiments. All data were fitted using a 1:1 binding model.

To also consider the issue of ligand depletion, we reduced the number of available interaction partners on the cell surface (i.e., CD19) by strongly decreasing the number of NALM6 cells in the reaction (tenfold reduced cell number). This low number of NALM6 cells was spiked into CD19-negative Jurkat cells, which do not participate in the binding reaction, but which facilitate the formation of a cell pellet in the centrifugation steps. The apparent *K*_D_ was observed to be even slightly lower when adapting experimental conditions to avoid ligand depletion (Suppl. Figure 1B vs. A). Together, when comparing standard experimental conditions (0.5 h incubation with a typical number of antigen-positive cells (1.5 × 10^5^) used in such assays) with an improved experimental condition (4 h incubation with an excess of ligand), we observed an eightfold difference in affinity (1.8 vs. 0.22 nM; Fig. 2A and Suppl. Figure 1A and B). Of note, these affinities were obtained by direct experimental comparison with identical interaction partners. Only the incubation time and the number of available cell surface antigens was varied. Thus, these results might—at least partially—explain the variations of FMC63-scFv affinities reported in literature.

### FMC63-scFv binds with comparable affinities to WT-CD19 and its stabilized variant SF-CD19

To analyze the FMC63-scFv affinity also in other experimental systems, soluble CD19 extracellular domain (CD19-ECD) is required. However, the CD19-ECD is known to be poorly expressed and unstable and to show a strong tendency to aggregate^20,21,25^, thus precluding reliable affinity analyses. Recently, we have developed stabilized versions (termed “SuperFolder”, SF) of the CD19-ECD^20^. In the present study, we used the variant SF05^20^, which will be termed SF-CD19 from hereon. This SF-CD19 contains only three point mutations located distant from the FMC63 epitope (Fig. 2C)^5,20,26^. In contrast to its wild type counterpart, SF-CD19 can be efficiently expressed in a monomeric and correctly folded state^20^. To investigate whether the three stabilizing point mutations affect the affinity to the FMC63-scFv, we expressed both WT-CD19 and SF-CD19 on Jurkat cells. Titration of the soluble FMC63-scFv yielded virtually identical affinities to these two CD19 variants (Fig. 2B). Thus, SF-CD19 can be used as a stable and monomeric reagent to assess the FMC63-scFv affinity and therefore SF-CD19 was utilized in the following experimental setups.

It should be noted that viability/stability constraints of the human cells limited the incubation times to 4 h. However, expression of SF-CD19 on yeast cells enabled 24 h incubations, which did not further increase the measured affinity, suggesting that a further prolongation beyond the 4 h incubation is not necessary to sufficiently approach equilibrium (Suppl. Fig. 3).

### Using soluble SF-CD19 and FMC63-based CAR T cells to minimize avidity effects

In the affinity analyses presented so far, the use of improved experimental conditions (prolonged incubation times and conditions to prevent ligand depletion) yielded *K*_D_ values that are at the lower end of the published values. Thus, we questioned whether the *K*_D_ values were underestimated in these experiments described above. One explanation for a potential underestimation of the *K*_D_ (i.e., overestimation of affinity) is the presence of avidity (Fig. 1E). Of note, scFvs are known to partially form dimers through “domain swapping” with neighboring scFvs, resulting in so-called diabodies that contain two binding sites^8,27–29^. These bivalent diabodies can cause avidity effects when analyzed against a surface immobilized antigen. When we analyzed our FMC63-scFv by size exclusion chromatography (SEC), we also observed a monomer/dimer equilibrium with approximately 20% dimers (Suppl. Fig. 2A). Of note, since this is a dynamic equilibrium, purification of the monomeric fraction was not successful.

Thus, to minimize potential avidity effects, we inverted the assay system. That is, we titrated soluble SF-CD19 on T cells expressing an FMC63-based CAR. When comparing improved experimental conditions (4 h incubation; ligand in excess) with suboptimal conditions (0.5 h incubation; limited amount of ligand), we yielded pronounced affinity differences (0.79 vs. 14 nM, respectively, Fig. 2D). Thus, similar to the NALM6 titrations described above, these data demonstrate the importance of sufficient incubation times and measures to avoid ligand depletion. However, the *K*_D_ values obtained with soluble SF-CD19 were generally higher than those yielded from NALM6 titrations with soluble scFv, suggesting that avidity effects mediated by scFv dimerization indeed led to an overestimation of affinities when tested on NALM6 cells. Taken together, we argue that the analysis with (1) soluble SF-CD19, (2) prolonged incubation times (4 h) and (3) conditions that prevent ligand depletion represents the most accurate condition, yielding an affinity of the CD19-FMC63 interaction of 0.79 nM (Fig. 2D).

### Surface plasmon resonance (SPR)

**Figure 3:**
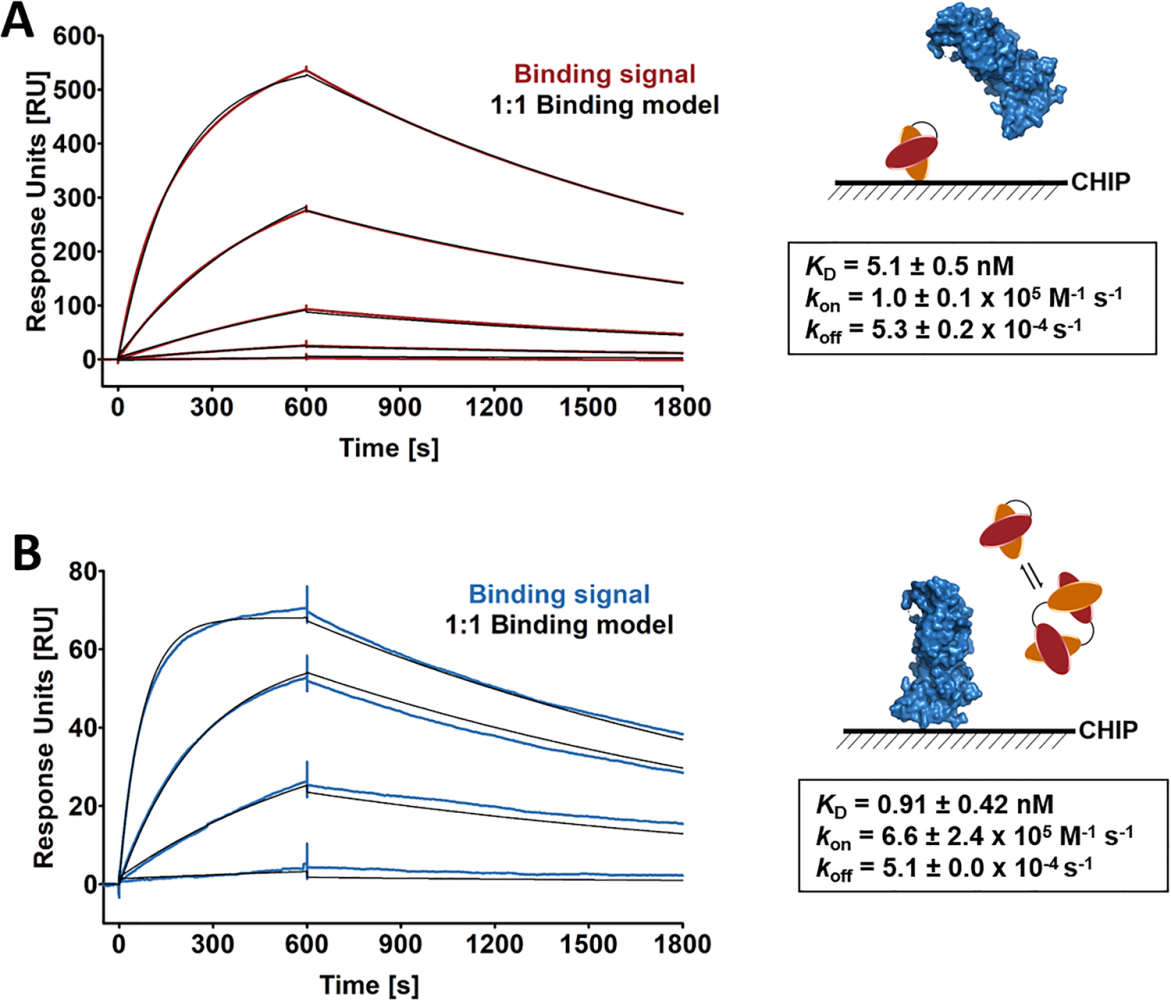
SPR measurements of binding kinetics between FMC63-scFv and tag-free SF-CD19. (**A**) SPR experiments were performed using a Biotin CAPture Chip S Series sensor chip. The FMC63-scFv was biotinylated and immobilized on the sensor chip. To collect kinetic binding data, tag-free SF-CD19 (> 99% monomer based on HPLC-SEC-MALS) was injected at 25 °C at a concentration of 40, 10, 2.5, 0.625 and 0.156 nM. (**B**) Tag-free SF-CD19 was biotinylated and immobilized on the sensor chip. Soluble FMC63-scFv was injected at 25 °C at a concentration of 40, 10, 2.5 and 0.156 nM. In (**A**, **B**) the complex was allowed to associate and dissociate for 600 and 1200 s, respectively. A kinetic 1:1 interaction model was fitted to the data using global data analysis. For the *K*_D_, *k*_on_ and *k*_off_, averages ± standard deviations of three independent experiments are shown.

Finally, we also performed surface plasmon resonance (SPR), which is often considered the gold standard for affinity measurements. For the cell titration experiments with SF-CD19 described above, we used a His_8_-SUMO-fusion construct, enabling detection with an anti-His-tag antibody. This fusion construct shows minor oligomerization (Suppl. Fig. 2B), albeit at a lower degree compared with the scFv, potentially also resulting in avidity effects. In contrast, SPR allows for label-free analysis with tag-free SF-CD19, which can be purified as a purely monomeric protein without any detectable oligomerization or aggregation (Suppl. Fig. 2C). Thus, we immobilized the FMC63-scFv on the chip surface, followed by injection of soluble, tag-free and monomeric SF-CD19. Fitting a kinetic 1:1 binding model to the binding curves yielded an affinity of 5.1 nM, as well as kinetic rate constants *k*_on_ (1.0 × 10^5^ M^−1^ s^−1^) and *k*_off_ (5.3 × 10^–4^ s^−1^) (Fig. 3A).

To directly test our hypothesis that scFv oligomerization causes avidity effects and therefore an affinity overestimation, we inverted the system. That is, tag-free SF-CD19 was immobilized on the SPR chip, followed by injection of soluble FMC63-scFv. This experimental setup yielded an affinity of 0.91 nM (Fig. 3B), i.e., a ~ sixfold higher affinity compared with the original, inverted setup, even though the exact same components were used. These data indicate that the use of soluble, partially dimeric scFv may result in an avidity-mediated overestimation of the affinity and therefore we argue that the SPR setup with soluble tag-free SF-CD19 (which is strictly monomeric) represents the more accurate experiment.

### Artefacts result in almost 100-fold variation of the FMC63 affinity

**Figure 4:**
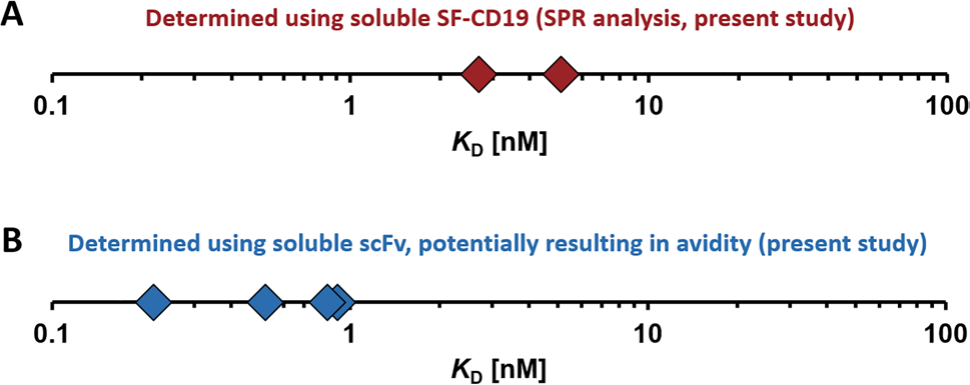
Overview of determined FMC63-scFv affinities (*K*_D_) to CD19. (**A**) Affinities determined in the present study using soluble tag-free SF-CD19 in SPR experiments (with the FMC63-scFv being immobilized on the chip surface). (**B**) Affinities determined in the present study using soluble FMC63-scFv in SPR and cell titration experiments. Values obtained from experiments with insufficient incubation times and/or conditions that promote ligand depletion are not shown here.

As extensively discussed above, the affinity of the FMC63-scFv can be influenced by a number of artefacts, including avidity effects, ligand-depletion, insufficient equilibration times and misfolded protein components, just to name a few. We provide strong evidence that these artefacts indeed affect the measured FMC63 affinities. We show with several different experimental setups that avidity effects lead to an overestimation of affinity, as suggested by the fact that assays with soluble scFv (which is partially dimeric) yield lower *K*_D_ values (Fig. 4B vs. A). On the other hand, insufficient incubation times and/or ligand depletion result in an underestimation of affinity, i.e. an artificially increased *K*_D_ (Fig. 2A,B,D, Suppl. Fig. 1). Our data show that even under standard experimental conditions (e.g. 30 min incubation, 1.5 × 10^5^ cells per tube, etc.) these artefacts caused variations by almost 100-fold (0.22–14 nM; Fig. 2). Thus, our observations also provide a potential explanation for the wide range of FMC63-scFv affinities reported in literature (Fig. 1A).

Alternatively, the wide range of published FMC63-scFv affinities may also be explained by the different scFv formats used. As shown in Fig. 1B, different groups used different scFv architectures, e.g., V_L _− V_H_ versus V_H _− V_L_ orientations, different linkers connecting the two variable domains and various fusion partners attached to the scFv, as well as different expression systems to produce the scFv. To address the possibility that the expression system and the scFv fusion partners strongly influence the obtained affinity, we also performed SPR measurements using an FMC63-scFv that was generated in a different laboratory (TU Munich). Apart from being expressed in another lab, this so-called FMC63-scFv FLEXamer was fused to other tags (a Twin-Strep-tag® and a Tub-tag)^30^ and it was also produced in a different expression system (*E. coli* instead of HEK293). In addition, we used a different SPR immobilization system for this FMC63-scFv FLEXamer. That is, the FMC63-scFv FLEXamer was immobilized on a Strep-Tactin®XT-coated CM5 chip via its Twin-Strep-tag. In contrast, in the SPR experiments described above, the FMC63-scFv was biotinylated and subsequently immobilized on a streptavidin-coated chip surface. Nevertheless, despite these differences, we obtained an affinity of 2.7 nM (Suppl. Fig. 4), which is highly comparable to that obtained with the HEK293-produced scFv fused to other peptide tags and anchored differently to the chip (5.1 nM, Fig. 3A). Thus, our data suggest that the variations of published FMC63-scFv affinities are mostly caused by experimental artefacts (e.g., ligand depletion, limited incubation time, avidity effects) rather than by different experimental setups and expression systems.

However, despite having different tags for purification and SPR immobilization and being expressed in different host organisms and in different laboratories, our two scFv versions contained identical V region orientations (V_L _− V_H_) and the same linker connecting the two variable domains and therefore potential influences of these parameters on the obtained affinity cannot be excluded.

### A discussion on the “true” FMC63 affinity

Given the artefacts discussed above, the correct *K*_D_ value can only be approached with an optimal experimental system. We argue that the SPR assays provided the best possible conditions and components, because (1) the soluble interaction partner (tag-free SF-CD19) is present in a strictly monomeric state, thus precluding avidity, (2) kinetic analysis eliminates the necessity to let the system approach equilibrium, (3) the constant flow on the SPR chip minimizes ligand-depleting effects and (4) both protein components were shown to be properly folded. Moreover, the fact that a global kinetic fit properly matched several curves obtained with a range of concentrations, also suggests that this system represents a defined 1:1 interaction without major artefacts.

Apart from SPR analysis, the only other experimental system not using soluble, partially dimeric FMC63-scFv (potentially causing avidity) was the CAR T cell titration assay. Importantly, even if the scFv also oligomerizes in the CAR constructs, this would not cause avidity effects as long as the soluble interaction partner (CD19) is monomeric. The *K*_D_ obtained with that titration setup (0.79 nM) was slightly lower as compared with the SPR assays. This minor variation may be explained by the fact that also the His_8_-SUMO-SF-CD19 fusion construct showed slight oligomerization and/or by the different experimental temperatures (4 °C for all cell titrations vs. 25 °C for SPR). As noted above, the tag-free version of SF-CD19 used for SPR experiments is virtually oligomer-free and therefore we consider the SPR experiments with soluble SF-CD19 to be the most reliable assay system in this study.

Summing up, we argue that the most reliable affinity values are those obtained from SPR experiments with soluble tag-free SF-CD19, which yielded affinities in the range of ~ 2–6 nM (at pH 7.4 and 25 °C).

## Conclusion

Despite its high importance in the CAR field, the affinity between the FMC63 benchmark scFv and its antigen CD19 has not been unambiguously determined. In this study, we not only determined the affinity of this important scFv with a broad range of experimental approaches, but we also provide potential explanations for the wide affinity range reported in literature. We demonstrate that various artefacts need to be taken into consideration during experimental design to be able to reliably measure this high affinity interaction. In addition, we also present SPR experiments with well-defined, monomeric proteins, thus also providing important kinetic rate constants of this key interaction. Since CAR affinity is known to be a major determinant for its function, we expect that this detailed affinity analysis of the FMC63 benchmark scFv will be highly valuable for interpretations of CAR affinity-function relationships, as well as for the design of future CAR T cell generations.

## Methods

### Cell culture

NALM6 and Jurkat cells were gifts from Dr. Sabine Strehl and Dr. Michael Dworzak, respectively (both at CCRI, Vienna, Austria). Cell culture experiments were approved by the Committee for Biological Safety of the Department of Biotechnology, University of Natural Resources and Life Sciences, Vienna. All experiments and methods were carried out in accordance with relevant guidelines and regulations.

NALM6, NALM6-GFP (generated in our previous study^17^) and Jurkat cells were maintained in RPMI-GlutaMAX (Thermo Scientific) supplemented with 10% fetal bovine serum (FBS, Sigma-Aldrich), 100 U/ml penicillin and 100 µg/ml streptomycin (Thermo Scientific) at 37 °C, 5% CO_2_ and a humidified atmosphere. HEK293T LentiX cells (Takara) were cultivated in DMEM (Thermo Scientific) supplemented with 4 mM L-glutamine (Thermo Scientific), 10% FBS, 100 U/ml penicillin and 100 µg/ml streptomycin. HEK293-6E cells (NRC Biotechnology Research Institute) were maintained in FreeStyle F17 expression medium (Thermo Fisher Scientific) supplemented with 4 mM L-glutamine, 25 µg/ml G418 (Biochrom) and 0.1% pluronic acid (Thermo Fisher) in plane, vented Erlenmeyer shaker flasks at 37 °C, 80% humidity, 8% CO_2_ and 130 rpm in a Climo-Shaker ISF1-XC (Adolf Kühner AG).

### Yeast surface display

Yeast cells displaying the SF-CD19 constructs were prepared as described previously^20,31^. Briefly, *Saccharomyces cerevisiae* (strain EBY100, ATCC) were transformed with the pCT-CON2 vector encoding the following construct: Aga2p—HA tag—(G_4_S)_3_ linker—SF-CD19 (Pro20-Pro278)—c-myc tag. For flow cytometric analysis, yeast cells were grown in SD-CAA medium (20 g/l glucose, 6.7 g/l yeast nitrogen base, 5 g/l casamino acids, 11.85 g/l sodium citrate dihydrate and 7.4 g/l citric acid monohydrate) over night at 30 °C while shaking. The following day, cultures were diluted to an OD_600_ of 0.2 in SD-CAA medium. At an OD_600_ of approx. 1, cells were centrifuged at 2000 g for 3 min, resuspended in the same volume of SG-CAA medium (20 g/l galactose, 2 g/l glucose, 6.7 g/l yeast nitrogen base, 5 g/l casamino acids, 10.2 g/l disodium hydrogen phosphate, 8.56 g/l sodium dihydrogen phosphate) and incubated over night at 20 °C while shaking to induce surface expression.

### Expression and purification of soluble SF-CD19 and FMC63-scFv

SF-CD19 and FMC63-scFv were expressed transiently in HEK293-6E cells. Both proteins were cloned into the pTT5 vector (NRC Biotechnology Research Institute). The vector incorporating the SF-CD19 insert was constructed as described previously^20^. The sequence encoding the His_8_-SUMO-SF-CD19 fusion protein is shown in the Supplementary Table 1. The FMC63-scFv expression vector was designed by cloning the coding sequence for the IgGк signal peptide in front of the variable domain of the light chain (V_L_) of the murine anti-CD19 clone FMC63, a Whitlow-linker^32^ and the variable domain of the heavy chain (V_H_) followed by a C-terminal His_8_ tag for purification and detection (sequence shown in the Supplementary Table 1). Transient transfection of HEK293-6E cells was performed at a cell density of 1–1.4 × 10^6^ cells/ml using 1 µg plasmid DNA and 2 µg PEI 20 k (1 mg/ml; Polysciences) per ml cell culture volume. Cells were fed with 0.5% (w/v) tryptone N1 (Organotechnie) and 1% glucose (Sigma-Aldrich) 48 h post transfection. Cell culture supernatant was harvested 5 days post transfection by two centrifugation steps, first at 300 g for 5 min to remove cells and afterwards at 16,000 g for 30 min to further clear the supernatant. Subsequently, cleared supernatant was filtered through an 0.45 µm PVDF filter (Merck) and concentrated using a Labscale TFF System equipped with a 10 kDa cutoff Pellicon TM device (Merck). The concentrated supernatant was supplemented with equal volume of 40 mM phosphate buffer containing 400 mM NaCl and 40 mM imidazole (pH 7.4). Purification was performed by applying the supernatant to a 5 ml HisTrap HP column (Cytiva) and bound protein was eluted with a linear gradient of 20–500 mM imidazole using an NGC chromotography system (Bio-Rad). Pooled fractions of the protein of interest were concentrated using Amicon Ultrafilters with a cutoff of 10 kDa (Merck). Buffer exchange to 20 mM phosphate buffer (pH 7.4) containing 200 mM NaCl was performed either with PD-10 columns (Cytiva) or overnight dialysis. Removal of fusion tags of the SF-CD19 protein was performed by overnight cleavage with 0.1 µg/ml precision HRV 3C protease at 4 °C. Tags and protease (both of which contained a His-tag) were removed by reverse HisTrap purification followed by size exclusion chromatography on a HiLoad 16/600 Superdex 200 pg column (Cytiva) equilibrated with a 20 mM phosphate buffer containing 200 mM NaCl (pH 7.4). Purified proteins were stored at − 80 °C until further use.

### Expression and purification of FMC63-scFv FLEXamer

The FMC63-scFv FLEXamer construct (DNA template of the scFv linked to a Twin-Strep-tag® and a Tub-tag sequence)^30^ was introduced into the pASG-IBAwt2 vector (IBA Lifesciences GmbH) according to the manufacturer’s protocol to enable periplasmic expression of the soluble protein (sequence shown in the Supplementary Table 1).

For the transformation of electrocompetent *E. coli* JM83 with the FMC63-scFv FLEXamer construct, 100 ng target DNA were inoculated into 200 µl bacteria solution, which was electroporated using a 1 mm Gene Pulser electroporation cuvette [1.8 kV, Pulse Controller and Gene Pulser (Bio-Rad)]. Electroporated bacteria were plated on Luria broth plates (1 µg/ml ampicillin) and incubated overnight at 37 °C. For the periplasmic expression of recombinant FMC63-scFv FLEXamers, one CFU was pre-cultured in 3 ml Luria broth liquid culture (1 µg/ml ampicillin) and grown at 37 °C under 200 rpm agitation. After 7 h of shaking, 1 l high-density medium (0.04% (w/v) yeast extract, 42 mM Na_2_HPO_4_, 51 mM KH_2_PO_4_, 10 mM NaOH, 24% (w/v) MgSO_4_*7 H_2_O, 0.7% (v/v) glycerin) was inoculated with the pre-culture, and bacteria were grown at 22 °C under 180 rpm agitation overnight. After reaching an optical density (OD_600_) of 3.0, periplasmic expression of the scFv FLEXamers was induced by adding anhydrotetracycline (final concentration of 0.2 µg/µl). After 3 h of protein expression, bacteria were centrifuged (5000 rpm, 4 °C, 12 min), and the pellets were stored at − 80 °C for further protein purification.

Strep-tag based protein purification was performed with gravity flow Strep-Tactin® Sepharose® columns (IBA Lifesciences GmbH) according to manufacturer’s instructions followed by a size exclusion chromatography on a HiLoad 16/600 Superdex 200 pg column (Cytiva) equilibrated with a 20 mM phosphate buffer containing 200 mM NaCl (pH 7.4). Purified proteins were stored at − 80 °C until further use.

### Determination of protein concentration

Concentrations of proteins used in the study were determined by UV absorption (280 nm) and a biuret-based method (Pierce™ Rapid Gold BCA Protein Assay Kit, Thermo Fisher). The following theoretical molecular weights (MW) and extinction coefficients were derived using the online ProtParam tool (Expasy, SIB Swiss Institute of Bioinformatics): His_8_-SUMO-SF-CD19: 43.79 kDa, 67,950 M^−1^ cm^−1^; tag-free SF-CD19: 28.31 kDa, 61,335 M^−1^ cm^−1^; FMC63-scFv: 26.54 kDa, 51,590 M^−1^ cm^−1^. Absorbance at 280 nm was measured using a DeNovix DS-11-Series (DeNovix). The BCA protein assay was performed according to the manufacturer’s protocol.

### Size-exclusion chromatography coupled to multi-angle light scattering

Size-exclusion chromatography coupled to multi-angle light scattering (SEC-MALS) was used to determine the molecular mass and aggregation state of recombinant SF-CD19 and FMC63-scFv. Analyses were performed on a high-performance liquid chromatography system (Shimadzu Prominence LC20) equipped with a MALS (Wyatt Heleos Dawn8 + plus QELS) and refractive index detector (RID-10A, Shimadzu). The column (Superdex™ 75 Increase 10/300 GL or Superdex™ 200 Increase 10/300 GL, GE Healthcare) was equilibrated with phosphate buffered saline (PBS) supplemented with 200 mM NaCl (pH 7.4) as running buffer. All proteins were filtered (0.1 µm Ultrafree-MC filter; Merck Millipore) before analysis and 25 µg of His_8_-SUMO-SF-CD19, tag-free SF-CD19, FMC63-scFv or FMC63-scFv FLEXamer were injected at a flow rate of 0.75 ml/min at 25 °C and analyzed using the ASTRA 6 software (Wyatt Technology) and LabSolutions software (Shimadzu). His_8_-SUMO-SF-CD19, tag-free SF-CD19 and FMC63-scFv were analyzed with a Superdex™ 200 Increase 10/300 GL column while FMC63-scFv FLEXamer was analyzed with Superdex™ 75 Increase 10/300 GL column.

### Biotinylation of tag-free SF-CD19 and FMC63-scFv

His-purified FMC63-scFv and cleaved SF-CD19 were biotinylated using the EZ-Link™ Sulfo-NHS-LC-Biotinylation Kit (Thermo Fisher) according to the manufacturer´s protocol.

### Surface plasmon resonance

Surface plasmon resonance experiments were performed using the Biacore T200 instrument (GE Healthcare). To collect kinetic binding data between tag-free SF-CD19 and FMC63-scFv, two different immobilization systems were applied. In a first setup, biotinylated FMC63-scFv or SF-CD19 (tag-free) were immobilized on a Biotin CAPture S Series sensor chip (Cytiva) at a flow rate of 10 µl/min and a concentration of 10 µg/ml yielding a density of approx. 1000 response units (RU) for both proteins. Next, SF-CD19 (tag-free) or FMC63-scFv were injected at a flow rate of 30 µl/min at 25 °C, followed by injection of buffer only. The complex was allowed to associate and dissociate for 600 and 1200 s, respectively. The chip surfaces were regenerated by injection of 3 M GuHCl + 1 M NaOH after each cycle for 120 s at a flow rate of 10 µl/min. These SPR experiments were performed in PBS supplemented with 0.1% bovine serum albumin (BSA, Sigma) and 0.05% Tween-20 (Sigma) (pH 7.4).

In a second setup, a CM5 sensor chip was coated with Strep-Tactin®XT. Twin-Strep-tagged FMC63-scFv FLEXamer was captured at a concentration of 50 nM and a contact time of 60 s. Next, tag-free SF-CD19 was injected at a flow rate of 30 µl/min at 25 °C. The complex was allowed to associate and dissociate for 600 and 1200 s, respectively. After each cycle the chip surfaces were regenerated by injection of 3 M GuHCl for 60 s at a flow rate of 10 µl/min. These SPR experiments were performed in HBS-EP Buffer (0.01 M HEPES (pH 7.4), 0.15 M NaCl, 3 mM EDTA and 0.005% v/v Surfactant P20, Cytiva).

For all SPR experiments, data were fit with a 1:1 kinetic binding model using the global data analysis option available within Biacore T200 Evaluation Software (GE Healthcare).

### Surface expression of WT-CD19 and SF-CD19 on Jurkat cells

Transgenes for in vitro transcription and subsequent transient CD19 surface expression were designed by cloning a T7 promoter upstream of the native signal peptide of CD19 (Uniprot P15391; amino acids 1–19), followed by a linker sequence (G_4_S), a Flag-tag, a linker sequence (G_4_S)_2_ and a truncated sequence of the native CD19 (Uniprot P15391; amino acids 20–317) containing the extracellular domains and the transmembrane domain (sequence shown in the Supplementary Table 1). SF-CD19 transgenes were generated by replacing the sequence between position Pro20 and Pro278 with the nucleotide sequence harboring the three stabilizing point mutations (M75V, R76S and F85S) by standard cloning techniques. DNA encoding the respective construct for WT-CD19 and SF-CD19 was amplified by PCR and transcribed in vitro with the mMessage mMachine T7 Ultra Kit (Thermo Fisher) according to the manufacturer’s instructions. Finally, mRNA was purified using the RNeasy Kit (Qiagen). Jurkat cells were transfected with 3 µg mRNA by electroporation of 5 × 10^6^ cells in 100 µl Opti-MEM (Thermo Scientific) using a square wave protocol (1 pulse, 500 V) for 3 ms. Electroporation was performed using 4 mm electroporation cuvettes (VWR) and the Gene Pulser Xcell Electroporation System (Bio-Rad). After electroporation, cells were immediately transferred to pre-warmed cell culture medium and used within 48 h after transfection for flow cytometric analysis.

### Generation of stable FMC63-41BBz CAR expressing Jurkat cells

To generate VSV-G pseudotyped lentivirus, Lenti-X 293 T cells (Takara) were co-transfected with a third generation puromycin-selectable pCDH expression vector (Systems Biosciences) containing the FMC63-41BBz CAR sequence and second-generation viral packaging plasmids pMD2.G and psPAX2 (Addgene plasmids #12,259 and #12,260, respectively). Briefly, the FMC63-scFv sequence was cloned in the V_L-_V_H_ orientation linked with a Whitlow-linker followed by a CD8α stalk and the transmembrane region (Uniprot P01732; amino acids 138–206), 4-1BB endodomain (Uniprot Q07011; amino acids 214–255) and the CD3ζ endodomain (Uniprot P20963-3; amino acids 52–164, Q65K). Lenti-X 293 T cells were transfected using PureFection Transfection Reagent (System Biosciences) and supernatants were collected on day 2 and 3 after transfection and concentrated using the Lenti-X Concentrator (Takara). Viral suspensions were resuspended in Jurkat cell culture medium and stored at − 80 °C. Jurkat cells were transduced by exposure of different final concentrations of lentiviral supernatants for 3 days, followed by selection with puromycin. Expression of the FMC63-41BBz CAR was verified by staining with SF-CD19 conjugated with AF-647 followed by flow cytometric analysis.

### Flow cytometric analysis—mammalian cells

Jurkat and NALM6-GFP cells were counted with disposable counting slides using a TC10 automated cell counter (Bio-Rad). Cells were washed with staining buffer (PBS supplemented with 0.1% BSA) and transferred to 96-well V-bottom plates (VWR). A total of 0.15 × 10^6^ cells were taken per well for flow cytometric analysis. Incubation with varying concentrations of either FMC63-scFv or His_8_-SUMO-SF-CD19 was performed for the indicated times (0.5, 1, 2 and 4 h) at 4 °C while shaking. Afterwards, cells were centrifuged at 300 g for 5 min. The supernatant was discarded and cells were washed with 200 µl of staining buffer followed by another centrifugation step at 300 g for 5 min. Binding of either FMC63-scFv or His_8_-SUMO-SF-CD19 was detected by staining the His-tag with α-HIS-AF647 (Qiagen) antibody at a final concentration of 4 µg/ml for 30 min at 4 °C and, finally, washed once. Subsequently, cells were analyzed on a CytoFLEX instrument (Beckman Coulter), followed by data analysis using the FlowJo Software (version 10.6.2). Non-transfected cells or unstained cells served as negative controls. Geometric mean fluorescence intensity (gMFI) of the binding signal was background-subtracted, fitted with a 1:1 binding model and normalized.

To avoid ligand depletion, staining conditions were adjusted by reducing the number of antigen positive cells. That is, 10% positive cells (expressing either CD19 or the FMC63-41BBz CAR, depending on the assay setup) were spiked with 90% negative cells. NALM6-GFP and Jurkat cells were gated according to their intracellular fluorescent marker proteins. Jurkat cells transiently expressing the membrane-anchored WT-CD19 and SF-CD19 versions were additionally stained via the Flag-tag expressed at the N-terminus of the CD19-constructs with α-Flag-PE at a final concentration of 4 µg/ml (Biolegend) in order to differentiate CD19 positive cells from CD19 negative cells. After gating on the cell population expressing the respective cell surface molecules (CD19 or the FMC63-41BBz CAR), they were analyzed for binding to the respective soluble ligand (FMC63-scFv or SF-CD19, respectively). That is, the 90% negative cells were only added to facilitate the formation of a cell pellet during centrifugation, but they did not participate in the binding reaction and they were not included in the final data analysis.

### Flow cytometric analysis—yeast cells

The yeast cell number was determined according to OD_600_ after induction of protein surface expression overnight. An OD_600_ of 1 corresponds to approximately 1 × 10^7^ cells/ml^31^. Staining of yeast cells was performed in PBS supplemented with 0.1% BSA. In total 1 × 10^6^ cells per well were used for flow cytometry. SF-CD19 expressing yeast were incubated with varying concentrations of His-tagged FMC63-scFv for 30 min or 24 h at 4 °C while shaking. Titration of FMC63-scFv was additionally performed by spiking SF-CD19-positive yeast with non-induced yeast cells to avoid potential ligand depletion at low ligand concentrations. After a washing step, a secondary labeling step was performed with 4 µg/ml of α-HIS-AF647 (Qiagen) and 2 µg/ml α-HA-AF488 for 30 min at 4 °C while shaking. After a final wash step, cells were analyzed on a CytoFLEX instrument (Beckman Coulter) and analyzed using the FlowJo Software (Version 10.6.2). Only SF-CD19-positive cells (as shown by HA-tag expression) were analyzed for FMC63-scFv binding.

### Supplementary Information

Supplementary Information.

## Data Availability

The data generated in this study are available upon request from the corresponding author.
