# Supplementary material for: Solving the mystery of the FMC63-CD19 affinity
Source: Sci Rep. 2023 Dec 27;13:23024. doi: 10.1038/s41598-023-48528-0 (PMC10754921; doi:10.1038/s41598-023-48528-0)
Supplement: Supplementary file 1 — Supplementary Information. [file 41598_2023_48528_MOESM1_ESM.pdf]

Supplementary Information

Solving the mystery of the FMC63-CD19 affinity

Jacqueline Seigner[1,2], Charlotte U. Zajc[1,3], Sarah Dötsch[4], Caroline Eigner[1], Elisabeth Laurent[5], Dirk H. Busch[4], Manfred Lehner[3,6,7], Michael W. Traxlmayr[1,3,*]

[1] Department of Chemistry, Institute of Biochemistry, University of Natural Resources and Life Sciences, Vienna, Austria

[2] Department of Biotechnology, Institute of Animal Cell Technology and Systems Biology, University of Natural Resources and Life Sciences, Vienna, Austria

[3] CD Laboratory for Next Generation CAR T Cells, Vienna, Austria

[4] Institute for Medical Microbiology, Immunology and Hygiene, Technical University of Munich, Munich, Germany

[5] BOKU Core Facility Biomolecular & Cellular Analysis, University of Natural Resources and Life Sciences, Vienna, Austria

[6] St. Anna Children´s Cancer Research Institute, CCRI, Vienna, Austria

[7] Department of Pediatrics, St. Anna Kinderspital, Medical University of Vienna, Vienna, Austria

*Correspondence: michael.traxlmayr@boku.ac.at

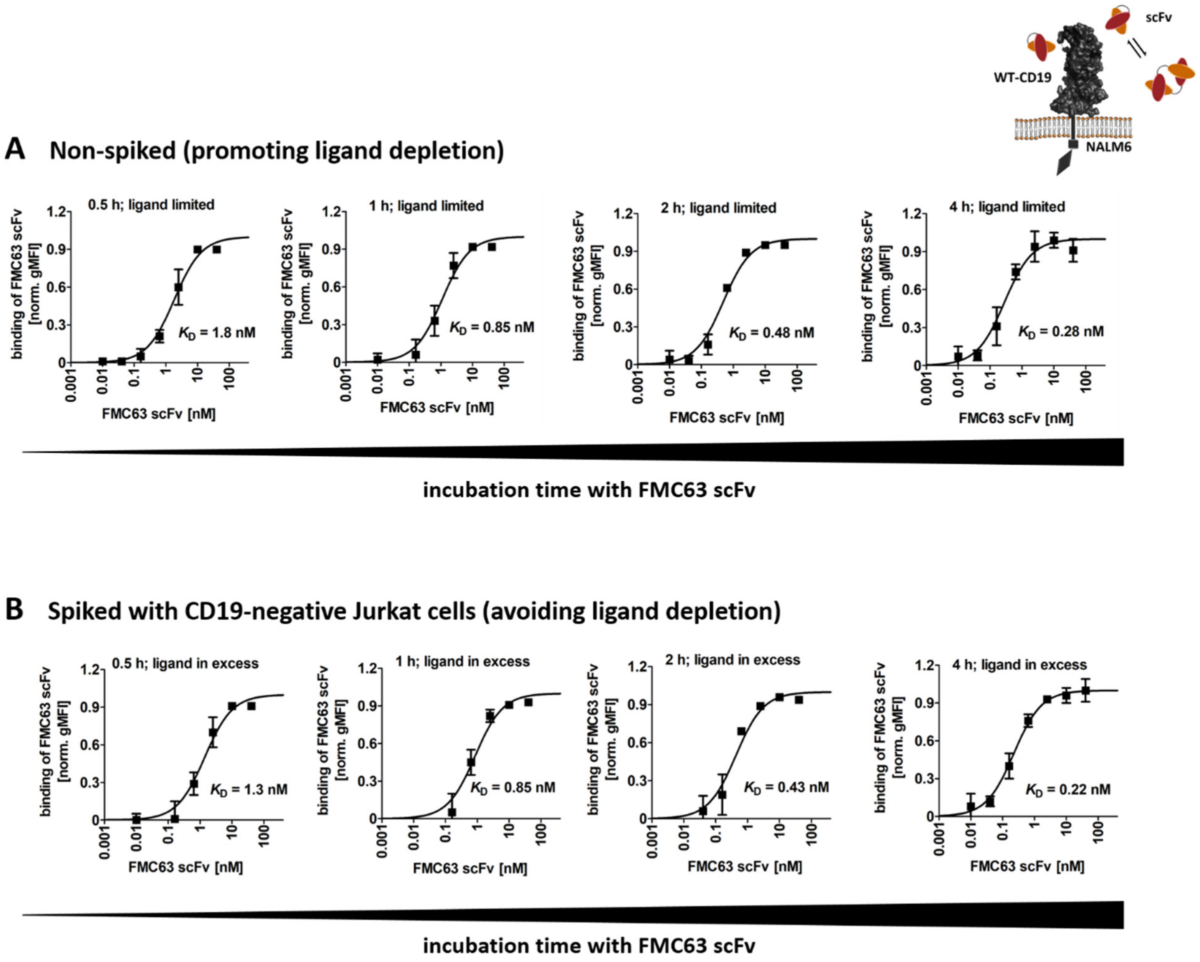

Supplementary Figure 1. The effect of ligand depletion and insufficient equilibration time in a binding assay.

(A) NALM6-GFP cells were incubated with various concentrations of soluble His-tagged FMC63-scFv at 4 °C for 0.5, 1, 2 or 4 h, as indicated.

(B) To avoid ligand depletion, 10% NALM6-GFP cells were spiked with 90% CD19-negative Jurkat cells. Cells were incubated with various concentrations of soluble His-tagged FMC63-scFv at 4 °C for 0.5, 1, 2 or 4 h, as indicated.

In **(A) and (B)** secondary staining was performed with α-HIS-AF647. The binding signal of the GFP-positive population was background subtracted, fitted with a 1:1 binding model and subsequently normalized. Shown are averages ± standard deviations of three independent experiments.

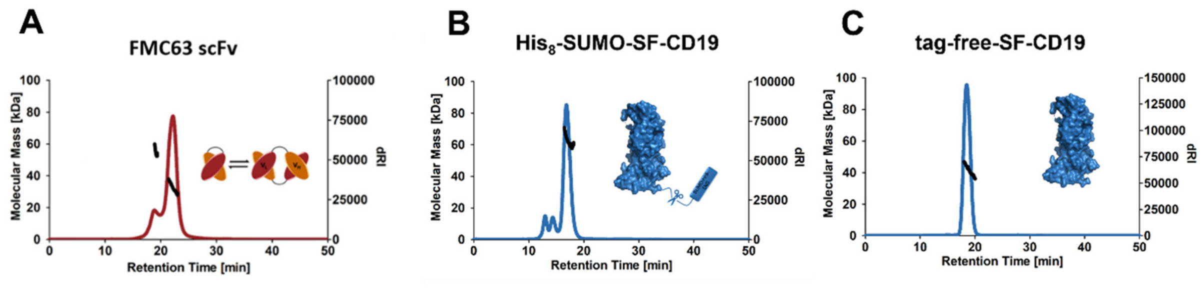

Supplementary Figure 2. Size exclusion chromatography profiles and molecular masses measured by MALS detection of **(A)** FMC63-scFv, **(B)** SF-CD19 fusion protein and **(C)** tag-free SF-CD19.

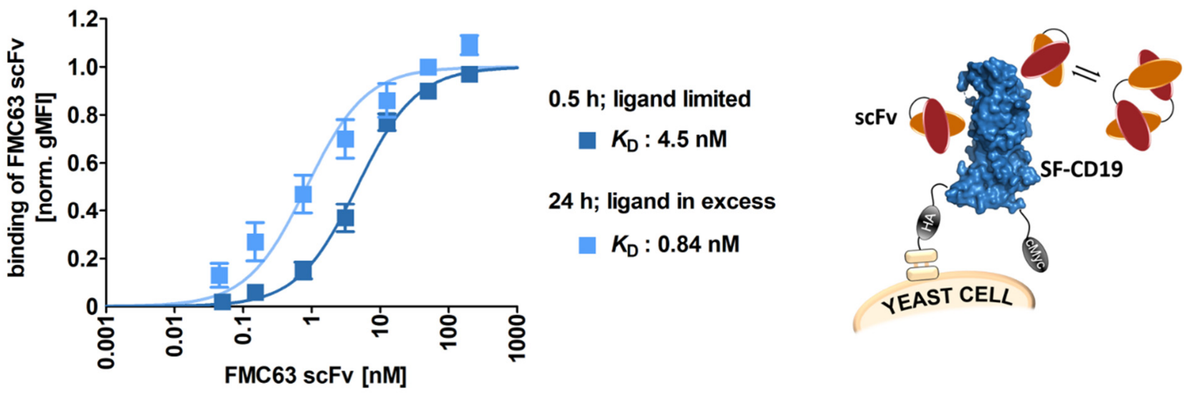

Supplementary Figure 3. Determination of binding affinity of FMC63-scFv to SF-CD19 using yeast surface display.

Yeast cells displaying SF-CD19 on their surface were incubated with various concentrations of His-tagged FMC63-scFv for 0.5 or 24 h at 4 °C. Staining conditions were chosen to either promote or avoid ligand depletion, as indicated. Secondary staining was performed with α-HIS-AF647 (to measure FMC63-scFv binding) and α-HA-AF488 (to measure surface expression of the SF-CD19 fusion construct). gMFI of the FMC63-scFv binding signal of the displaying (i.e., HA-positive) population was background-subtracted, fitted with a 1:1 binding model and normalized. Shown are averages ± standard deviations of three independent experiments.

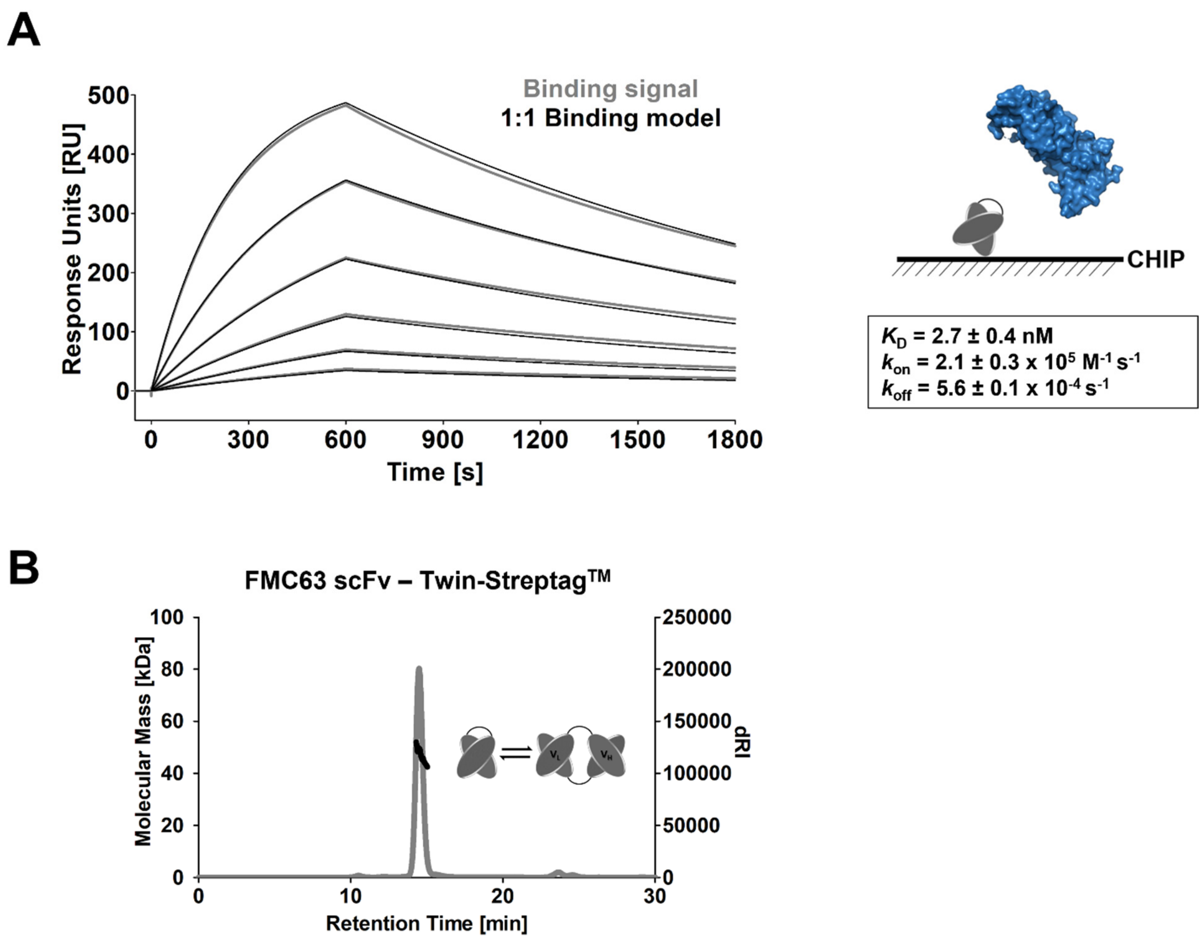

Supplementary Figure 4. SPR measurements of binding kinetics between FMC63-scFv FLEXamer and tag-free SF-CD19.

(A) SPR experiments were performed using a CM5 sensor chip coated with Strep-Tactin®XT. FMC63-scFv FLEXamer was immobilized at a concentration of 50 nM at an injection rate of 10 µl/min for 60 s. Soluble and tag-free SF-CD19 was injected at a flow rate of 30 µl/min at a concentration of 40, 13.3, 4.4, 1.5, 0.49 and 0.16 nM. The complex was allowed to associate and dissociate for 600 and 1200 s, respectively. Data were fitted with a kinetic 1:1 interaction model using the global data analysis option available within Biacore T200 Evaluation Software.

(B) Size exclusion chromatography profile and molar mass of the FMC63-scFv FLEXamer according to MALS detection.

CONSTRUCT	DESIGN	AMINO ACID SEQUENCE
**WT-CD19 surface expression (membrane bound)**	**Signal peptide $_{CD19}$ -G_4S-linker – Flag - 2(G_4S)-linker – WT-CD19 (proline 20 – leucin 317)**	MPPPRLLFFLLFLTPMEVRGGGGSDYKDDDDKGGGGSGGGGSPEEPLVV<br>KVEEGDNAVLQCLKGTSDGPTQQLTWSRESPLKPFLKLSLGLPGLGIHMRP<br>LAIWLFIFNVSQQMGGFYLCQPGPPSEKAWQPGWTVNVEGSGELFRWN<br>VSDLGGLGCGLKNRSSEGPSSPSGKLMSPKLYVWAKDRPEIWEGEPPCLPP<br>RDSLNQSLSQDLTMAPGSTLWLSCGVPPDSVSRGPLSWTHVHPKGPKSLL<br>SLELKDDRPARDMWVMETGLLLPRATAQDAGKYYCHRGNLTMSFHLEITA<br>RPVLWHWLLRTGGWKVSAVTLAYLIFCLCSLVGILHLQRAL*
**SF-CD19 surface expression (membrane bound)**	**Signal Peptide $_{CD19}$ -G_4S-Linker – Flag - 2(G_4S)-Linker – SF-CD19 (proline 20 – leucin 317)**	MPPPRLLFFLLFLTPMEVRGGGGSDYKDDDDKGGGGSGGGGSPEEPLVV<br>KVEEGDNAVLQCLKGTSDGPTQQLTWSRESPLKPFLKLSLGLPGLGIHVSPL<br>AIWLFISNVSQQMGGFYLCQPGPPSEKAWQPGWTVNVEGSGELFRWNV<br>SDLGGLGCGLKNRSSEGPSSPSGKLMSPKLYVWAKDRPEIWEGEPPCLPPR<br>DSLNQSLSQDLTMAPGSTLWLSCGVPPDSVSRGPLSWTHVHPKGPKSLLS<br>LELKDDRPARDMWVMETGLLLPRATAQDAGKYYCHRGNLTMSFHLEITAR<br>PVLWHWLLRTGGWKVSAVTLAYLIFCLCSLVGILHLQRAL*
**SF-CD19 fusion protein expression (soluble)**	**Signal Peptide $_{\text{Ig}\kappa\ \text{Leader}}$ – His_8 - AviTag™ – SUMO – HRV 3C site – SF-CD19 (proline 20 – Proline 278)**	METDTLLLWVLLLWVPGSTGDGHHHHHHHHGLNDIFEAQKIEWHEGSL<br>QDSEVNQEAKPEVKPEVKPETHINLKVSDGSSEIFFKIKKTTPLRRLMEAFA<br>KRQGKEMDSLTFLYDGIEIQADQTPEDLDMEDNDIIEAHREQIGGGSLEVL<br>FQGPPEEPLVVKVEEGDNAVLQCLKGTSDGPTQQLTWSRESPLKPFLKLSL<br>GLPGLGIHVSPLAIWLFISNVSQQMGGFYLCQPGPPSEKAWQPGWTVNV<br>EGSGELFRWNVSDLGGLGCGLKNRSSEGPSSPSGKLMSPKLYVWAKDRPE<br>IWEGEPPCLPPRDSLNQSLSQDLTMAPGSTLWLSCGVPPDSVSRGPLSWT<br>HVHPKGPKSLLSLELKDDRPARDMWVMETGLLLPRATAQDAGKYYCHRG<br>NLTMSFHLEITARP*
**FMC63 scFv (soluble)**	**Signal peptide – V_L – Whitlow-linker – V_H - G_4S-linker – His_8**	MELGLSWIFLLAILKGVQCDIQMTQTTSSLSASLGDRVTISCRASQDISKYLN<br>WYQQKPDGTVKLLIYHTSRLHSGVPSRFSGSGSGTDYSLTISNLEQEDIATYF<br>CQQGNTLPYTFGGGTKLEITGSTSGSGKPGSGEGSTKGEVKLQESGPGLVA<br>PSQSLSVTCTVSGVSLPDYGVSWIRQPPRKGLEWLGVIWGSETTYYNSALK<br>SRLTIIKDNSKSQVFLKMNSLQTDDTAIYYCAKHYYYGGSYAMDYWGQGTS<br>VTVSSGGGGSHHHHHHHH*
**FMC63 scFv – Twin-Streptag™ (soluble)**	**V_L – Whitlow-linker – V_H – GHGSGSA – Strep-TagII – Linker – Strep-TagII**	MDIQMTQTTSSLSASLGDRVTISCRASQDISKYLNWYQQKPDGTVKLLIYH<br>TSRLHSGVPSRFSGSGSGTDYSLTISNLEQEDIATYFCQQGNTLPYTFGGGT<br>KLEITGSTSGSGKPGSGEGSTKGEVKLQESGPGLVAPSQSLSVTCTVSGVSLP<br>DYGVSWIRQPPRKGLEWLGVIWGSETTYYNSALKSRLTIIKDNSKSQVFLK<br>MNSLQTDDTAIYYCAKHYYYGGSYAMDYWGQGTSVTVSSGHGSGSAWS<br>HPQFEKGGGSGGGSGGSAWSHPQFEKVDSVEGEGEEEGEE*
**FMC63 scFv based chimeric antigen receptor (membrane bound)**	**Signal peptide – V_L – Whitlow-linker – V_H - 3xAlanine – $CD8\alpha_{Hinge}$ – $CD8\alpha_{Transmembrane}$ – $4\text{-}1BB_{Endoomain}$ – $CD3\zeta_{Signaling\ domain}$**	MLLLVTSLLLCELPHPAFLLIPDIQMTQTTSSLSASLGDRVTISCRASQDISKYL<br>NWYQQKPDGTVKLLIYHTSRLHSGVPSRFSGSGSGTDYSLTISNLEQEDIAT<br>YFCQQGNTLPYTFGGGTKLEITGSTSGSGKPGSGEGSTKGEVKLQESGPGL<br>VAPSQSLSVTCTVSGVSLPDYGVSWIRQPPRKGLEWLGVIWGSETTYYNSA<br>LKSRLTIIKDNSKSQVFLKMNSLQTDDTAIYYCAKHYYYGGSYAMDYWGQG<br>TSVTVSSAAATTTPAPRPPTPAPTIASQPLSLRPEACRPAAGGAVHTRGLDF<br>ACDIYIWAPLAGTCGVLLLSLVITLYCKRGRKKLLYIFKQPFMRPVQTTQEED<br>GCSCRFPEEEEGGCELRVKFSRSADAPAYKQGQNQLYNELNLGRREEYDVL<br>DKRRGRDPEMGGKPRRKNPQEGLYNELQKDKMAEAYSEIGMKGERRRG<br>KGHDGLYQGLSTATKDTYDALHMQALPPR
**SF-CD19 yeast surface expression (cell surface bound)**	**Ag2P – HA tag - 3(G_4S)-linker – SF-CD19 (proline 20 – proline 278) – GS – c-myc tag**	MQLLRCFSIFSVIASVLAQELTTICEQIPSPTLESTPYSLSTTTILANGKAMQG<br>VFEYYKSVTFVSNCGSHPSTTSKGSPINTQYVFKDNSSTIEGRYPYDVPDYAL<br>QASGGGGSGGGGSGGGGSASPEEPLVVKVEEGDNAVLQCLKGTSDGPTQ<br>QLTWSRESPLKPFLKLSLGLPGLGIHVSPLAIWLFISNVSQQMGGFYLCQPG<br>PPSEKAWQPGWTVNVEGSGELFRWNVSDLGGLGCGLKNRSSEGPSSPSG<br>KLMSPKLYVWAKDRPEIWEGEPPCLPPRDSLNQSLSQDLTMAPGSTLWLS<br>CGVPPDSVSRGPLSWTHVHPKGPKSLLSLELKDDRPARDMWVMETGLLL<br>PRATAQDAGKYYCHRGNLTMSFHLEITARPGSEQKLISEEDL*

Supplementary Table 1. Amino acid sequences of protein constructs used in this study.
